# Avaliação da Relação entre Níveis de Adropina e Circulação Colateral Coronária em Pacientes com Síndrome Coronariana Crônica

**DOI:** 10.36660/abc.20210573

**Published:** 2022-06-23

**Authors:** Hasan Akkaya, Ertuğrul Emre Güntürk, Fulya Akkaya, Uğur Karabıyık, İnayet Güntürk, Samet Yılmaz

**Affiliations:** 1 Departamento de Cardiologia Universidade Niğde Ömer Halisdemir Hospital Acadêmico e de Pesquisa da Faculdade de Medicina Niğde Turquia Departamento de Cardiologia, Universidade Niğde Ömer Halisdemir, Hospital Acadêmico e de Pesquisa da Faculdade de Medicina, Niğde – Turquia; 2 Associação de Hospitais Públicos do Ministério da Saúde Niğde Turquia Associação de Hospitais Públicos do Ministério da Saúde, Niğde – Turquia; 3 Departamento de Bioquímica Universidade Niğde Ömer Halisdemir Escola de Saúde Zübeyde Hanım Niğde Turquia Departamento de Bioquímica, Universidade Niğde Ömer Halisdemir, Escola de Saúde Zübeyde Hanım, Niğde – Turquia

**Keywords:** Síndrome Coronariana Aguda, Aterosclerose, Peptídeos, Adropina, Coronariopatias, Circulação Colateral Coronária, Diagnóstico por Imagem, Angiografia Coronária

## Abstract

**Fundamento:**

A circulação colateral coronária (CCC) proporciona um fluxo sanguíneo alternativo a tecido miocárdico exposto a isquemia e ajuda a preservar as funções miocárdicas. A produção endotelial de óxido nítrico (NO) e o fator de crescimento endotelial vascular (VEGF) foram apontados como os fatores mais importantes no desenvolvimento da CCC. A adropina é um hormônio peptídeo responsável pela hemostasia energética, e é conhecida por seus efeitos positivos no endotélio por NO e VEGF.

**Objetivo:**

O objetivo deste estudo é investigar a associação entre adropina e a presença de CCC em pacientes com síndrome coronariana crônica (SCC)

**Métodos:**

Um total de 102 pacientes com SCC, que tinham oclusão total de pelo menos 1 artéria coronária epicárdica importante, foram incluídos no estudo e foram divididos em dois grupos: o grupo de pacientes (n: 50) com CCC ruim (Rentrop 0-1) e o grupo de pacientes (n: 52) com CCC boa (Rentrop 2-3). O nível de significância adotado para a análise estatística foi 5%.

**Resultados:**

Os níveis médios de adropina identificados foram 210,83±17,76 pg/mL e 268,25±28,94 pg/mL nos grupos com CCC ruim e boa, respectivamente (p<0,001). Detectou-se que os níveis de adropina têm correlação com as razões neutrófilo-linfócito (r: 0,17, p: 0,04) e com os escores de Rentrop (r: 0,76, p<0,001), e correlação negativa com idade (r: -0,23, p: 0,01) e com os escores Gensini (r: -0,19, p: 0,02). O nível de adropina é um preditor independente da boa evolução da CCC (RC: 1.12, IC 95%: (1,06–1,18), p<0,001).

**Conclusão:**

Este estudo sugere que os níveis de adropina podem ser um fator associado à de CCC em pacientes com SCC.

## Introdução

A doença arterial coronariana (DAC) é uma doença caracterizada pelo estreitamento ou a oclusão das artérias coronárias, geralmente devido a aterosclerose. Ela é a principal causa de morte em homens e mulheres em todo o mundo, e sua incidência aumenta com a idade.^1^ Na síndrome coronariana crônica (SCC), os sintomas podem variar com o tempo devido a fatores como consumo de oxigênio miocárdico, stress emocional, ou variações de temperatura. Além disso, a SCC está associada à estabilidade ou quiescência da placa aterosclerótica.^1^

A adropina é um hormônio peptídeo composto de setenta e seis aminoácidos que é codificada pelo “gene associado à homeostase energética (ENHO)”. O termo “adropina” derivou-se das palavras em Latim “aduro” e “pinquis”, se refere a um agente que promove a queima de gordura.^2^ Os efeitos da adropina nas doenças cardíacas foram sugeridas por vários mecanismos. Entretanto, seus efeitos nas funções endoteliais foram aceitos como seu principal mecanismo. A adropina aumenta a expressão de eNOS, que é o responsável principal pela produção de NO. Paralelamente, a deficiência de adropina foi associada à redução da biodisponibilidade de NO endotélio.^3^ Além disso, relatou-se que a adropina inibe a agregação de plaquetas,^4^ proliferação do músculo liso,^5^ adesão endotelial de leucócitos e monócitos^6^ e oxidação do LDL.^7^ A disfunção endotelial caracterizada pela deficiência de NO endotelial é um preditor independente do surgimento de DAC. Sabe-se que a adropina é eficiente no metabolismo de NO. Concordantemente, foram demonstrados seus efeitos positivos nas funções endoteliais,^8^ e baixos níveis de adropina foram associados a disfunção endotelial.^8 , 9^ Além disso, demonstrou-se que pacientes com síndrome X têm níveis mais baixos de adropina em comparação a indivíduos saudáveis.^10^

A adropina ativa o receptor 2 do fator de crescimento endotelial vascular (VEGFR-2) e vias de fosfatidilinositol-3-fosfato quinase no endotélio da parede do vaso, e contribui para a secreção do óxido nítrico (NO) aumentando a atividade da sintase endotelial do óxido nítrico (eNOS). A literatura relata que a adropina levou indiretamente à vasodilatação na parede do vaso, e que a injeção de adropina sintética em um tecido, no qual a isquemia se desenvolveu, levou à cura do tecido por reperfusão.^8^

A literatura também demonstra que o desequilíbrio entre o suprimento de oxigênio miocárdico e a demanda de oxigênio resultante de estenose da artéria coronária ou oclusão da artéria coronária aumenta a evolução de circulação colateral coronária (CCC). A formação de CCC ocorre na forma de “angiogênese”, que ocorre novamente pelo surgimento de novos capilares a partir de vasos sanguíneos existentes, ou “arteriogênese”, que ocorre como resultado do crescimento e da maturação de canais de anastomose que existem entre as artérias existentes desde o nascimento.^11^

A tecnologia atual não permite a medição não invasiva de CCC em seres humanos. Portanto, a maneira mais fácil de se avaliar a CCC é pela avaliação visual das artérias colaterais usando-se a angiografia coronária, que pode ser feite em um método semiquantitativo, conforme descrito por Rentrop et al.^12^

Há muitos estudos disponíveis na literatura sobre os fatores que afetam a CCC. Entretanto, não estudos em que o efeito dos níveis de adropina na CCC tenham sido tratados, apesar de haver vários estudos conduzidos em anos anteriores que demonstraram a função protetiva da adropina na estrutura e função endoteliais. Considerando o mencionado acima, pela primeira vez, neste estudo, a adropina é investigada quanto à possibilidade de ser um fator associado à presença de CCC do ponto de vista patofisiológico em indivíduos portadores de SCC.

## Métodos

Foram incluídos neste estudo 102 pacientes que passaram por AC devido a SCC entre março de 2017 e março de 2020 em Niğde Ömer Halisdemir University Hospital (centro único), prospectivamente. Os pacientes foram divididos em dois grupos: o grupo de pacientes com CCC ruim (Rentrop 0-1) (n: 50) e o grupo de pacientes com CCC boa (Rentrop 2-3) (n: 52), com base em escores de Rentrop.

Pacientes com SCC, que tinham oclusão total de pelo menos uma artéria coronária epicárdica na angiografia coronária, foram incluídos no estudo, enquanto pacientes que tiveram síndrome coronária aguda nos últimos 6 meses, operação de by-pass de artéria coronária anterior (CABG), doença de válvula cardíaca moderada a grave, insuficiência renal aguda/crônica, níveis de TFG (taxa de filtração glomerular estimada) <30 ml/min, insuficiência hepática, qualquer malignidade conhecida, sintomas de insuficiência cardíaca [NYHA (New York Heart Association) classe 3 ou 4], obstrução pulmonar crônica moderada/grave, qualquer doença infecciosa aguda/crônica, e doenças inflamatória ou reumatológica aguda/crônica, foram excluídos deste estudo.

Pacientes cujos níveis de pressão arterial identificados foram >140/90 mm/Hg resultantes de medições repetidas ou que foram identificados como usuários de medicamentos anti-hipertensivos foram considerados pacientes portadores de hipertensão, enquanto que pacientes cujos níveis de glicemia plasmática em jejum identificados foram >126 mg/dL como resultados de medições repetidas ou que foram identificados como usuários de medicamentos antidiabetes foram considerados pacientes portadores de diabetes mellitus.

As amostras de sangue foram coletadas de forma venosa depois de pelo menos 10 horas de jejum, e, em seguida, foram centrifugadas rapidamente a 1000 g e 4 °C por 10 minutos. O soro sanguíneo resultante foi armazenado a -80 °C para análise bioquímica. As concentrações de adropina foram estudadas duas vezes usando um kit ELISA comercialmente disponível (Fankew, Shanghai Kexing Trading Co., Ltd, China). Identificou-se que os coeficientes inter- e intraensaio da variação estavam abaixo de 9% e 10% respectivamente.

Todos os pacientes passaram por ecocardiograma transtorácico realizado pelo mesmo cardiologista, e sua fração de ejeção ventricular esquerda (FEVE) foi calculada pelo método de Simpson.

Os valores de índice de massa corporal (IMC) (kg/m^2^ ) dos pacientes foram calculados dividindo-se seus pesos por suas alturas ao quadrado.

### Avaliações angiográficas

As imagens angiográficas foram avaliadas por dois cardiologistas experientes usando os sistemas Picture Archiving and Communication. Dois cardiologistas tomaram uma decisão conjunta no caso de lesões limítrofes.

Os escores de Gensini foram calculados com base no grau de estenose angiográfica. Dessa forma, foi atribuído 1 ponto para estenose 0-25%, foram atribuídos 2 pontos para estenose 25-50%, foram atribuídos 4 pontos para estenose 50-75%, foram atribuídos 8 pontos para estenose 75-90%. Foram atribuídos 16 pontos para estenose 90-99%, e foram atribuídos 32 pontos para lesão de 100% (oclusão total). Esses escores foram multiplicados pelo coeficiente definido para cada artéria coronária principal e todos os pontos de segmento [artéria descendente anterior esquerda: 5, segmento proximal da artéria descendente anterior (ADA): 2,5, segmento médio da ADA: 1,5, segmento apical da ADA: 1, primeiro ramo diagonal: 1, segundo ramo diagonal: 0,5, segmento proximal da artéria circunflexa (Cx) na presença da dominância da artéria coronária direita (ACD): 2,5, segmento distal da artéria Cx: 1, ramo marginal obtuso: 1, ramos póstero-lateral: 0,5, segmento proximal da ACD: 1, segmento médio da ACD: 1, segmento dista da ACD: 1, e artéria descendente posterior: 1].^13^

A classificação de Rentrop é feita com base na angiografia coronária. Assim, casos sem fluxo colateral da artéria coronária com um fluxo sanguíneo, para a artéria completamente ocluída, foram avaliadas como grau 0, casos com preenchimento nos ramos laterais da artéria ocluída, mas sem enchimento no seguimento epicárdico, foram avaliados como grau 1, casos com enchimento parcial do segmento epicárdico foram avaliados como grau 2, e casos com enchimento colateral total do vaso epicárdico foram avaliados como grau 3.^12^

### Análise estatística

O pacote do software SPSS 23.0 (Statistical Package for the Social Sciences Version 23.0) foi usado para conduzir as análises estatísticas. O teste de Kolmogorov-Smirnov foi usado para avaliar o padrão de distribuição dos dados de pesquisa. Variáveis numéricas com distribuição normal foram expressas em termos de média ± desvio padrão (DP)), enquanto variáveis numéricas sem distribuição normal foram expressas em termos de mediana e faixa interquartil (FIQ). As variáveis categóricas foram resumidas como números e porcentagens e comparadas entre os grupos usando-se o teste qui-quadrado. As variáveis que apresentaram distribuição normal entre os grupos foram comparadas usando-se o teste t de Student não pareado, e aquelas não têm distribuição normal foram comparadas usando-se o teste U de Mann Whitney. Um valor de p<0,05 foi considerado estatisticamente significativo. Análises de regressão logística univariada e multivariada foram realizadas para identificar preditores independentes de CCC boa. O teste de correlação de Spearman foi realizado para definir a correlação entre o nível de adropina e outros parâmetros. A curva de característica de operação do receptor (ROC) foi usada para revelar a sensibilidade, a especificidade e o valor de corte ideal do nível de adropina que pode ser usado para prever a CCC boa.

## Resultados

Um total de 102 pacientes, dos quais 50 tinham CCC ruim e 52 tinham CCC boa, foram incluídos no estudo. Nenhuma diferença significativa foi encontrada entre os grupos de pacientes com CCC ruim ou boa com relação a sexo, idade, IMC, condição de tabagismo, diabetes mellitus (DM), hipertensão, níveis de pressão arterial, frequências cardíacas, FEVE e medicamentos usados ( Tabela 1 ).

**Tabela 1:** Características clínicas da população do estudo

	CCC ruim (n: 50)	CCC boa (n: 52)	Valor de p
Masculino, n (%)	35 (70)	38 (73)	0,80
Idade, anos, média (DP)	60,47 (8,06)	59,04 (8,96)	0,47
IMC, média (DP), kg/m^2^	24,28 (1,61)	24,12 (1,62)	0,67
Fumante, n (%)	15 (30)	26 (50)	0,11
DM, n (%)	15 (30)	16 (31)	0,94
Hipertensão, n (%)	10 (20)	13 (25)	0,79
Pressão arterial sistólica, média (DP), mm Hg	122,77 (10,32)	125,87 (11,24)	0,22
Pressão arterial diastólica, média (DP), mm Hg	74,37 (8,50)	74,65 (8,63)	0,88
Frequência cardíaca, média (DP), bat/min	76,93 (13,95)	76,69 (13,61)	0,94
FEVE, (%), média (DP)	55,80 (8,18)	53,77 (8,12)	0,28
Uso de estatinas, n (%)	10 (20)	11 (21,1)	0,78
Uso de β-bloqueadores, n (%)	11 (22,2)	13 (25)	0,88
Uso de nitrato, n (%)	3 (6)	3 (5,7)	0,93
Uso de inibidores da enzima conversora de angiotensina, n (%)	9 (18)	8 (15,3)	0,74
Uso de bloqueador de receptor de angiotensina, n (%)	11 (22,2)	7 (13,5)	0,22
Uso de bloqueador dos canais de cálcio, n (%)	10 (20)	7 (13,5)	0,53

*CCC: circulação colateral coronária; DP: desvio padrão; IMC: índice de massa corporal; DM: diabetes mellitus; FEVE: fração de ejeção ventricular esquerda.*

As características laboratoriais dos grupos são apresentadas na Tabela 2 . Os níveis de adropina identificados foram significativamente diferentes, em 210,83±17,76 pg/mL e 268,25±28,94 pg/mL nos grupos com CCC ruim e boa, respectivamente. Os dois grupos não variaram significativamente em nenhum dos outros parâmetros laboratoriais.

**Tabela 2:** Características laboratoriais da população do estudo

	CCC ruim (n: 50)	CCC boa (n: 52)	Valor de p
Nível de adropina, média (DP), pg/mL	210,83 (17,76)	268.25 (28,94)	<0,001
Proteína C reativa de alta sensibilidade, mediana (FIQ), mg/L	3,55 (0,93)	3,40 (0,93)	0,68
Glicemia jejum, média (DP), mg/dl	117,03 (33,83)	123,80 (44,09)	0,48
Hemoglobina glicada, média (DP), %	6,40 (1,19)	6,39 (1,02)	0,97
Colesterol total, média (DP), mmol/L	187,90 (25,30)	194,23 (28,34)	0,31
HDL, média (DP), mmol/L	43,73 (5,57)	44,33 (6,17)	0,67
Triglicérides, mediana (IQR), mmol/L	154,00 (50,85)	163,50 (47,20)	0,73
LDL, média (DP), mmol/L	108,50 (28,20)	110,92 (29,60)	0,72
Creatinina, média (DP), mg/dL	1,14 (0,15)	1,13 (0,16)	0,71
Hemoglobina, média (DP), g/L	14,56 (0,94)	14,25 (1,09)	0,19
Largura de distribuição de glóbulos vermelho, média (DP), %	12,79 (1,01)	12,61 (1,14)	0,47
Leucócitos, média (DP), x 10^9^/L	8,34 (1,65)	8,36 (1,44)	0,95
Neutrófilos, média (DP), x 10^9^/L	6,31 (1,94)	6,21 (1,44)	0,78
Linfócitos, média (DP), x 10^9^/L	1,75 (0,44)	1,70 (0,43)	0,57
NLR, média (DP), %	3,92 (1,64)	3,89 (1,56)	0,76
Plaqueta, média (DP), x 10^9^/L	241,90 (41,35)	225,04 (39,80)	0,07

*CCC: circulação colateral coronária; DP: desvio padrão; NLR: razões neutrófilo-linfócito.*

As características angiográficas coronárias dos grupos de pacientes são apresentadas na Tabela 3 . Não foram encontradas diferenças significativas entre os grupos de acordo com a localização das artérias coronárias ocluídas. Os escores Gensini médios dos grupos com CCC ruim e boa identificados foram significativamente diferentes, em 104,3±18,9 e 95,3±14,4, respectivamente. Também não houve diferenças entre os grupos em termos da doença arterial coronariana principal esquerda, doenças multivasculares e lesões de bifurcação.

**Tabela 3:** Achados angiográficos coronários da população do estudo

	CCC ruim (n: 50)	CCC boa (n: 52)	Valor de p
Oclusão de ADA, n (%)	15 (30)	12 (23,1)	0,51
Oclusão de Cx, n (%)	15 (30)	16 (30,8)	0,93
Oclusão de ACD, n (%)	18 (36)	24 (46,2)	0,44
Escore de Gensini, média (DP)	104,3 (18,9)	95,3 (14,4)	0,007
Doença arterial coronariana esquerda principal, n (%)	3 (6)	2 (3,8)	0,08
Doença multivascular, n (%)	23 (46)	21 (40,4)	0,65
Lesões de bifurcação, n (%)	10 (20)	8 (15,4)	0,14
Escore de Rentrop 0, n (%)	18 (36)		
Escore de Rentrop 1, n (%)	32 (64)		
Escore de Rentrop 2, n (%)		31 (59,6)	
Escore de Rentrop 3, n (%)		21 (40,4)	

*CCC: circulação colateral coronária; ADA: artéria descendente anterior; Cx: artéria circunflexa; ACD: artéria coronária direita; DP: desvio padrão.*

**Figura 1:**
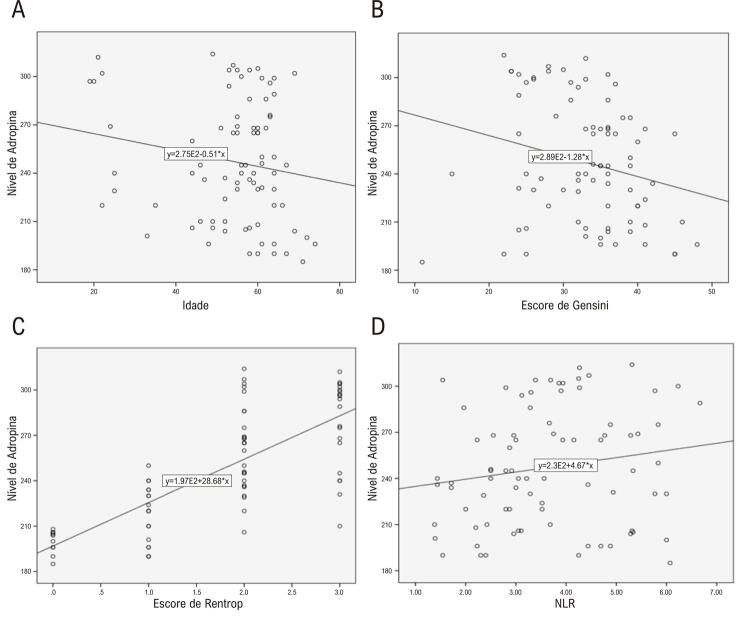
Gráficos de dispersão mostrando a relação entre níveis de adropina e a) Idade (r: -0,23, p: 0,01); b) Escore de Gensini (r: -0,19, p: 0,02); c) Escore de Rentrop (r: 0,76, p: <0,001); d) NLR (r: 0,17, p: 0,04).

Nenhuma correlação significativa foi encontrada entre os níveis de adropina e os valores de IMC, frequências cardíacas, níveis de proteína C reativa de alta sensibilidade, níveis de hemoglobina glicada (HbA1c), condição de tabagismo, presença de DM, presença de hipertensão, colesterol total, níveis de HDL, triglicérides e LDL. Observou-se uma correlação significativa e moderadamente positiva entre os níveis de adropina e as razões neutrófilo-linfócito (NLR); no entanto, observou-se uma correlação significativa e fortemente positiva entre os níveis de adropina e os escores de Rentrop. Por outro lado, observou-se uma correlação significativa e moderadamente negativa entre os níveis de adropina e idade e escores de Gensini ( Tabela 4 ) ( Figura 1 ).

**Tabela 4:** Correlação entre nível de adropina e outras variáveis da população do estudo

	r	Valor de p
Idade	-0,23	0,01
IMC	-0,10	0,55
Frequência cardíaca	0,12	0,43
Proteína C reativa de alta sensibilidade	0,04	0,84
Hemoglobina glicada	0,69	0,56
Fumante	0,33	0,16
DM	0,06	0,85
Hipertensão	0,09	0,51
Colesterol total	0,10	0,81
HDL	-0,14	0,45
Triglicérides	0,25	0,34
LDL	0,09	0,76
NLR	0,17	0,04
Escore de Gensini	-0,19	0,02
Escore de Rentrop	0,76	<0,001

*IMC: índice de massa corporal; DM: diabetes mellitus; NLR: razões neutrófilo-linfócito.*

**Figura 2:**
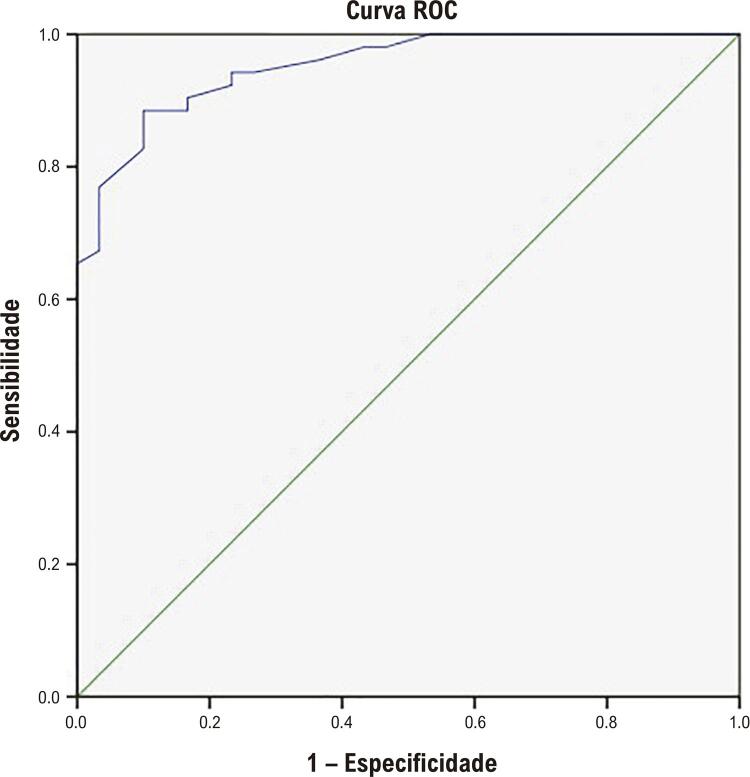
Análise de característica de operação do receptor (ROC) quanto ao nível de adropina para prever boa circulação colateral coronária.

Foi realizada a análise de curva ROC para avaliar o papel do nível de adropina na previsão de CCC boa ( Figura 2 ). A análise de ROC revelou que um valor de corte de 276,25 pg/mL em termos de nível de adropina previu a CCC boa com sensibilidade de 91% e especificidade de 96% (área ROC = 952, p<0,001).

Conforme mostrado na Tabela 2 , os níveis de adropina eram mais altos no grupo com CCC boa, e, portanto, foram realizadas análises para determinar se o nível de adropina pode ser usado como preditor independente da evolução de boa CCC. Os resultados da análise de regressão logística univariada indicaram que o nível de adropina era um fator independente e forte da evolução da CCC boa. Identificou-se que score de Gensini, doença multivascular, oclusão de ADA e oclusão de ACD eram preditores independentes da evolução da CCC boa também. Além disso, os resultados da análise de regressão logística multivariada, que foi ajustada quanto aos possíveis fatores de confusão, tais como, IMC, frequência cardíaca, colesterol total, e lipoproteína de baixa densidade (LDL), revelaram que não só os níveis de adropina, mas também o escore de Gensini, a doença multivascular, oclusão de ADA e oclusão de ACD eram preditores independentes da evolução da CCC boa ( Tabela 5 ).

**Tabela 5:** Análise de regressão logística univariada e multivariada demonstrando preditores independentes de CCC boa

	Univariada		Multivariada*	
	Valor de p	RC (IC 95%)	Valor de p	RC (IC 95%)
Nível de adropina	<0,001	1,12 (1,06-1,18)	<0,001	1,13 (1,06-1,19)
Idade	0,48	1,01 (0,97-1,04)	0,54	1,01 (0,97-1,05)
IMC	0,38	1,24 (0,76-2,02)	0,45	1,23 (0,74-2,01)
Frequência cardíaca	0,43	1,03 (0,98-1,05)	0,51	1,04 (1,01-1,07)
Proteína C reativa de alta sensibilidade	0,19	0,58 (0,26-1,30)	0,41	0,61 (0,30-1,42)
Hemoglobina glicada	0,96	0,98 (0,51-1,90)	0,34	1,01 (0,56-1,96)
Fumante	0,12	1,86 (0,89-3,89)	0,48	1,35 (0,55-3,32)
DM	0,81	1,09 (0,55-2,14)	0,87	1,06 (0,51-2,25)
Hipertensão	0,89	0,96 (0,48-1,90)	0,80	0,91 (0,43-1,93)
Colesterol total	0,09	1,42 (1,10-1,83)	0,10	1,44 (1,09-1,90)
HDL	0,48	1,56 (0,46-5,32)	0,85	1,05 (0,27-4,20)
Triglicérides	0,10	1,23 (0,96-1,58)	0,27	1,19 (0,88-1,60)
LDL	0,77	0,99 (0,97-1,02)	0,23	1,01 (0,98-1,03)
NLR	0,23	0,66 (0,44-1,10)	0,31	0,74 (0,51-1,33)
Escore de Gensini	<0,001	1,02 (1,01-1,03)	<0,001	1,01 (1,00-1,02)
Doença multivascular	<0,001	2,63 (1,68-4,14)	<0,001	2,45 (1,53-3,93)
Oclusão de ADA	<0,001	4,59 (2,13-9,90)	<0,001	4,73 (2,08-10,70)
Oclusão de Cx	0,09	2,21 (1,07–4,34)	0,11	2,41 (1,12-4,41)
Oclusão de ACD	0,01	2,31 (1,17-4,53)	0,03	2,17 (1,03-4,56)

*Ajustada quanto a idade, frequência cardíaca, IMC, colesterol total e LDL. Nessa análise estatística, os níveis de adropina, bem como idade, IMC, colesterol total, HDL, triglicérides, LDL, doença multivascular e escore de Gensini são valores contínuos, outros são variáveis binárias. *IMC: índice de massa corporal; DM: diabetes mellitus; NLR: razões neutrófilo-linfócito; ADA: artéria descendente anterior; Cx: artéria circunflexa; ACD: artéria coronária direita.*

## Discussão

Este é o primeiro estudo em que a relação entre níveis de adropina e CCC foi investigada em pacientes diagnosticados com SCC. O principal achado deste estudo foi que os níveis de adropina eram mais baixos no grupo com CCC ruim que no grupo com CCC boa. Além disso, observou-se uma correlação positiva entre os níveis de adropina e os valores de NLR e escores de Rentrop; no entanto, observou-se uma correlação negativa entre os níveis de adropina e idade e escores de Gensini. Ademais, a regressão logística e as análises de ROC indicaram que a adropina era um preditor independente da evolução de CCC boa. Além do nível de adropina, identificou-se que outros fatores, tais como, score de Gensini, presença de doença multivascular, oclusão de ADA e oclusão de ACD eram preditores independentes da evolução da CCC boa também.

A CCC ocorre quando os vasos coronários se estreitam em 70% ou mais.^14^ Os vasos colaterais resultantes têm tamanho entre 20-200 µm e paredes finas. A densidade dos vasos colaterais formados varia entre espécies, e é moderada nos seres humanos.^15^ Esses vasos são a via alternativa de suprimento de sangue para o miocárdio isquêmico. Os vasos de CCC geralmente são fechados e não funcionais. Entretanto, quando ocorre a diferença de pressão como resultado da estenose coronária, os vasos rudimentares se abrem rapidamente.^14^

As artérias coronárias colaterais ajudam a manter as funções miocárdicas, oferecendo um fluxo sanguíneo alternativo ao tecido miocárdico isquêmico esquerdo por DAC oclusiva. Geralmente é a isquemia que ocasiona um excesso de artérias colaterais. Entretanto, mesmo aquelas sem DAC têm artérias colaterais em excesso, já que a CCC existente pode se mostrar insuficientes durante o exercício, apesar de fornecer o sangue necessário para o miocárdio em repouso. Várias variáveis angiográficas de clínicas independentes foram associadas ao grau de CCC na literatura. Em pacientes com DAC, o momento da oclusão,^16^ a localização da lesão, a gravidade da estenose coronária, e a duração da angina^17^ afetam o grau de CCC; enquanto, em indivíduos saudáveis, a hipertensão e a frequência cardíaca em repouso^18^ afetam o grau de CCC;

A importância clínica da CCC é que ela protege as funções miocárdicas,^19^ limita a dimensão do infarto^20^ e afeta positivamente a remodelação ventricular,^21^ particularmente durante o infarto agudo do miocárdio. Além disso, já se relatou na literatura que a CCC reduziu parcialmente a incidência de choque cardiogênico concomitante.^22^

Recentemente, sugeriu-se que os fatores mais importantes na evolução da CCC são produção endotelial de NO e VEGF. Sabe-se que NO e VEGF aumentam a angiogênese, especialmente em vasos colaterais coronários, e contribuem para a maturação das artérias coronárias.^23^ Demonstrou-se que a adropina aumenta VEGFR-2 em células endoteliais, e, como resultado, demonstrou-se que ela também aumenta a expressão de mRNA eNOS e proteína eNOS, via Akt (transformação da cepa Ak), que é quinase proteína B, e ERK½ (quinase proteína regulada por sinal extracelular ½) também.^8^ Portanto, fica óbvio que as colaterais coronárias vão amadurecer mais por VEGFR-2. Na realidade, neste estudo clínico, observou-se uma correlação positiva e significativa entre os escores de Rentrop, o que indica colaterais coronárias, e os níveis de adropina, corroborando os achados do estudo celular mencionado acima.

Demonstrou-se uma relação entre DAC e níveis baixos de adropina; e entre os escores SYNTAX (do inglês *SYNergy between percutaneous coronary intervention with TAXus and cardiac surgery* - Sinergia entre intervenção coronária percutânea com Taxus e cirurgia cardíaca), de Gersini e de Friesinger e níveis séricos de adropina apresentaram uma correlação negativa nos pacientes do grupo com DM tipo 2.^24^ Sugere-se que níveis baixos de adropina sérica são um preditor independente da aterosclerose coronária^24^ e a patência dos enxertos venosos após a operação de CABG.^25^ Em comparação, neste estudo, de forma similar aos achados de outros estudos mencionados acima, uma correlação moderadamente negativa foi identificada entre os scores de Gensini e os níveis de adropina. Entretanto, neste estudo, pacientes com DM tipo 2 representavam 30,5% (30% no grupo com CCC ruim, 31% no grupo com CCC boa) do grupo do estudo. Além disso, os pacientes que passaram por CABG não foram incluídos neste estudo.

Vários estudos sugeriram que há uma relação inversa entre envelhecimento e níveis de adropina, e que essa redução dos níveis de adropina pode ser um dos fatores pequenos de desencadeiam DAC, cujo aumento com a idade não é conhecido.^9 , 24^ Outro estudo também demonstrou que o efeito de vasodilatação mediada por eNOS e induzida por adropina diminui com a idade.^26^ Comparativamente, neste estudo, assim como em achados de outros estudos mencionados acima, observou-se uma correlação significativa moderada entre níveis de adropina e idade na direção negativa.

Não há dúvidas de que a NLR está associada à inflamação e que a inflamação tem um papel nas DAC. Para dar um exemplo, um estudo realizados com pacientes de SCC crônica, a NLR média foi identificada como 5,0±5,1 no grupo com progressão de aterosclerose, e como 3,2±3,0 no grupo sem progressão, e esse achado foi atribuído à correlação entre progressão de aterosclerose e aumento da NLR.^27^ Além dos fatores de risco clássicos associados, a NLR demonstrou estar associada à prevalência de DAC bem como à complexidade das lesões.^28^ Em outro estudo controlado, valores altos de NLR demonstraram ser um bom preditor de escores de Gensini no grupo de pacientes com SCC. Valores de NLR acima de 2,04 demonstraram ter previsto eficientemente a presença de DAC.^29^ Na verdade, outro estudo demonstrou que valores de NLR previram as oclusões crônicas totais dos pacientes.^30^ Foi relatada uma correlação entre evolução de CCC boa e NLR no grupo de pacientes com oclusão total crônica concomitante.^31^ Em comparação, neste estudo, ao contrário aos achados respectivos relatados na literatura, não se identificou que a NLR foi diferente entre os grupos com CCC ruim e boa, porém detectou-se uma correlação com níveis de adropina. Considera-se que essa discrepância entre tal resultado deste estudo e os respectivos resultados relatados na literatura se devem ao baixo número de pacientes incluídos neste estudo.

### Limitações do estudo

Há várias limitações a este estudo. Primeiramente, o número de pacientes incluídos neste estudo foi limitado, e, segundo, não houve grupo de controle composto de indivíduos com artérias coronárias normais. Portanto, seria benéfico replicar o estudo com um grupo de estudo maior e com a adição de um grupo de controle. Além disso, a evolução da CCC é um longo processo, e, portanto, uma única medição dos níveis de adropina pode não dar uma ideia clara sobre o desenvolvimento de CCC vitalícia. Outra limitação foi que foi usada a classificação de Rentrop, um método visual usado na avaliação da CCC, e a ultrassonografia intravascular não foi usada. A CCC examinada na classificação de Rentrop é afetada pela pressão arterial do paciente, pela força de injeção de contraste do operador, e o tempo de filmagem. Por último, apesar de que se tenha identificado uma correlação entre os níveis de adropina e a CCC, os mecanismos subjacentes não estão claros, e, portanto, são necessários estudos de grande escala para verificar o efeito da adropina na evolução da CCC.

## Conclusão

Em conclusão, os achados deste estudo sugerem que os níveis de adropina estão correlacionados à presença de CCC em pacientes com SCC.
