# Supplementary material for: Avaliação da Relação entre Níveis de Adropina e Circulação Colateral Coronária em Pacientes com Síndrome Coronariana Crônica
Source: Arq Bras Cardiol. 2022 Jun 23;119(3):402–10. [Article in Portuguese] doi: 10.36660/abc.20210573 (PMC9438532; doi:10.36660/abc.20210573)
Supplement: Translation

# Translation: Assessment of the Relatıonshıp Between the Adropın Levels and the Coronary Collateral Cırculatıon in Patıents wıth Chronıc Coronary Syndrome

**Authors:** Hasan Akkaya, Ertuğrul Emre Güntürk, Fulya Akkaya, Uğur Karabıyık, İnayet Güntürk, Samet Yılmaz

**Affiliations:** 1 Turkey Cardiology Department, Niğde Ömer Halisdemir University Medicine Faculty Education and Research Hospital, Niğde – Turkey; 2 Turkey Ministry of Health Public Hospitals Association, Niğde – Turkey; 3 Turkey Biochemistry Department, Niğde Ömer Halisdemir University Zübeyde Hanım School of Health Niğde, Niğde – Turkey

**Keywords:** Acute Coronary Syndrome, Atherosclerosis, Peptides, Adropin, Coronary Artery Diseases, Coronary Collateral Circulation, Diagnostic Imaging, Coronary Angiography

## Abstract

**Background:**

Coronary collateral circulation (CCC) provides an alternative blood flow to myocardial tissue exposed to ischemia and helps to preserve myocardial functions. Endothelial-derived nitric-oxide (NO) production and vascular endothelial growth factor (VEGF) have been suggested as the most important factors in the development of CCC. Adropin is a peptide hormone responsible for energy hemostasis, and is known for its positive effects on the endothelium through NO and VEGF.

**Objective:**

The aim of this study is to investigate the association between adropin and the presence of CCC in patients with chronic coronary syndrome (CCS).

**Methods:**

A total of 102 patients with CCS, who had complete occlusion of at least one major epicardial coronary artery, were included in the study and were divided into two groups: the group of patients (n:50) with poor CCC (Rentrop 0-1) and the group of patients (n:52) with good CCC (Rentrop 2-3). The level of significance adopted in the statistical analysis was 5%.

**Results:**

Mean adropine levels were found as 210.83±17.76 pg/mL and 268.25±28.94 pg/mL in the poor and good CCC groups, respectively (p<0.001). Adropin levels proved to be positively correlated with neutrophil-to-lymphocyte ratios (r:0.17, p:0.04) and the rentrop scores (r:0.76, p<0.001), and negatively correlated with age (r:-0.23, p:0.01) and Gensini scores (r:-0.19, p:0.02). Adropin level is a strong independent predictor of good CCC development (OR:1.12, 95% CI:(1.06–1.18), p<0.001).

**Conclusion:**

This study suggests that adropin levels may be a possible factor associated with the presence of CCC in CCS patients.

## Introductıon

Coronary artery disease (CAD) is a disease characterized by narrowing or occlusion of the coronary arteries, usually due to atherosclerosis. It is the leading cause of death in men and women worldwide, and its incidence increases with age.^1^ In chronic coronary syndrome (CCS), symptoms may vary over time due to such factors as myocardial oxygen consumption, emotional stress, or temperature changes. CCS is also associated with the stability or quiescence of the atherosclerotic plaque.^1^

Adropin is a peptide hormone containing seventy-six amino acids and is encoded by the “energy homeostasis-associated gene (ENHO)”. The term “adropin” was derived from the Latin words of “aduro” and “pinquis”, and refers to an agent that promotes burning of fats.^2^ The effects of adropin in heart diseases have been suggested by various mechanisms, but its effects on endothelial functions have been accepted as its main mechanism. Adropin increases the expression of eNOS, which is primarily responsible for NO production. In parallel, adropin deficiency has been associated with a decrease in NO bioavailability in the endothelium.^3^ Furthermore, adropin has been reported to inhibit platelet aggregation,^4^ smooth muscle proliferation^5^ endothelial adhesion of leukocytes and monocytes,^6^ and LDL oxidation.^7^ Endothelial dysfunction characterized by endothelial NO deficiency is an independent predictor of the onset of CAD. Adropin is known to be effective on NO metabolism. Concordantly, its positive effects on endothelial functions have been shown,^8^ and low adropin levels have been associated with endothelial dysfunction.^8 , 9^ Additionally, Cardiac Syndrome X patients with endothelial dysfunction were shown to have lower adropin levels when compared to healthy individuals.^10^

Adropin activates the vascular endothelial growth factor receptor-2 (VEGFR-2) and phosphatidyl inositol-3-phosphate kinase pathways in the vessel wall endothelium, and contributes to the nitric oxide (NO) secretion by increasing the endothelial nitric oxide synthase (eNOS) activity. It has been reported in the literature that adropin indirectly led to vasodilation in the vessel wall, and that injection of synthetic adropin into a tissue, in which ischemia has developed, led to the healing of tissue through reperfusion.^8^

It has also been shown in the literature that the imbalance between myocardial oxygen supply and oxygen demand resulting from coronary artery stenosis or coronary artery occlusion increases the development of coronary collateral circulation (CCC). The formation of CCC occurs in the form of either “angiogenesis”, which occurs de novo through the budding of new capillaries from the existing blood vessels, or “arteriogenesis”, which occurs as a result of the growth and maturation of anastomosis channels that exist between the existing arteries since birth.^11^

Current technology does not allow the non-invasive measurement of CCC in humans. Thus, the easiest way to evaluate CCC is through the visual evaluation of the collateral arteries using coronary angiography, which can be done in a semi-quantitative method, as described by Rentrop et al.^12^

There are many studies available in the literature on the factors that affect the CCC. Nevertheless, there is no study in which the effect of adropin levels on the CCC has been addressed, despite the fact that there are a number of studies conducted in previous years which demonstrated the protective role of adropin on endothelial structure and function. In view of that mentioned above, for the first time, in this study, adropin is investigated as to whether it can be a possible factor associated with the presence of CCC from the pathophysiological standpoint in individuals with CCS.

## Methods

This study included, prospectively, 102 patients, who underwent CA due to CCS between March 2017 and March 2020 at Niğde Ömer Halisdemir University Hospital (Single-center). The patients were divided into two groups: the group of patients with poor CCC (Rentrop 0-1)(n:50) and the group of patients with good CCC (Rentrop 2-3)(n:52) based on the Rentrop scores.

Patients with CCS, who had complete occlusion of at least one major epicardial coronary artery on the coronary angiography, were included in the study, whereas patients who presented an acute coronary syndrome in the last 6 months, previous coronary artery bypass (CABG) operation, moderate to severe heart valve disease, acute/chronic kidney failure, eGFR (estimated glomerular filtration rate) levels of <30 ml/min, liver failure, any known malignancy, heart failure symptoms [NYHA (New York Heart Association) class 3 or 4], moderate/severe chronic obstructive pulmonary disease, any acute/chronic infective disease, and acute/chronic rheumatological or inflammatory disease, were excluded from the study.

Patients, whose blood pressure levels were found to be >140/90 mm/Hg as a result of repetitive measurements or who were found to have been using any antihypertensive medication, were considered to be hypertension patients, whereas patients, whose fasting plasma glucose levels were found to be > 126 mg/dL as a result of repetitive measurements or who were found to have been using any antidiabetic medication, were considered to be diabetes mellitus patients.

Blood samples were collected venously after at least 10 hours of fasting, and were then quickly centrifuged at 1000 g and 4°C for 10 minutes. The resultant blood serums were stored at -80°C for biochemical analysis. Serum adropin concentrations were studied twice, using a commercially available ELISA kit (Fankew, Shanghai Kexing Trading Co., Ltd, China). The inter-assay and intra-assay coefficients of variation were found to be below 9% and 10%, respectively.

All patients underwent a transthoracic echocardiography by the same cardiologist, and their left ventricular ejection fractions (LVEF) were calculated using the Simpson’s method.

Body mass index (BMI) (kg/m^2^ ) values of the patients were calculated by dividing their body weights by the squares of their heights.

### Angiographic evaluations

Angiographic images were evaluated by two experienced cardiologists using the Picture Archiving and Communication Systems. Two cardiologists made a joint decision in the case of borderline lesions.

Gensini scores were calculated based on the degree of angiographic stenosis. Accordingly, 1 point was assigned for 0-25% stenosis, 2 points were assigned for 25-50% stenosis, 4 points were assigned for 50-75% stenosis, 8 points were assigned for 75-90% stenosis, 16 points were assigned for 90-99% stenosis, and 32 points were assigned for 100% lesion (complete occlusion). These scores were then multiplied by the coefficient defined for each main coronary artery and each segment points [left main coronary artery:5, proximal segment of the left anterior descending artery (LAD):2.5, middle segment of LAD:1.5, apical segment of LAD:1, first diagonal branch:1, second diagonal branch:0.5, proximal segment of the circumflex artery (Cx) in the presence of right coronary artery (RCA) dominance:2.5, distal segment of the Cx artery:1, the obtuse marginal branch:1, posterolateral branch:0.5, RCA proximal segment:1, RCA middle segment:1, RCA distal segment:1, and posterior descending artery:1].^13^

Rentrop classification is made based on the coronary angiography. Accordingly, cases with no collateral flow from the coronary artery with a blood flow, to the completely occluded coronary artery were assessed as grade 0, cases that filled in the lateral branches of the occluded artery but that did no fill in the epicardial segment were assessed as grade 1; cases with partial filling in the epicardial segment were assessed as grade 2; and cases with complete collateral filling of the epicardial vessel were assessed as grade 3.^12^

### Statistical analysis

SPSS 23.0 (Statistical Package for the Social Sciences Version 23.0) software package was used to conduct the statistical analyses. Kolmogorov-Smirnov test was used to assess the distribution pattern of the research data. Normally distributed numerical variables were expressed in terms of mean± standard deviation (SD), whereas non-normally distributed numerical variables were expressed in terms of median and interquartile range (IQR). Categorical variables were summarized as numbers and percentages, and compared between the groups using the Chi-square test. The variables that showed normal distribution between the groups were compared using the unpaired Student’s t-test and those without a normal distribution were compared using the Mann-Whitney U-test. A value of p<0.05 was accepted as statistically significant. Univariate and multivariate logistic regression analyses were performed to identify the dependent predictors of good CCC. The Spearman correlation test was performed to define the correlation between adropin level and other parameters. Receiver operating characteristic (ROC) curve was used to reveal the sensitivity, specificity, and the optimal cut-off value of adropin level that can be used to predict good CCC.

## Results

A total of 102 patients, of whom 50 presented poor CCC and 52 presented good CCC, were included in the study. No significant differences were found between the patient groups with poor or good CCC in terms of gender, age, BMI, smoking status, diabetes mellitus (DM), hypertension, arterial blood pressure levels, heart rates, LVEF, and medications used ( Table 1 ).

**Table 1:** Clinical characteristics of the study population

	Poor CCC (n:50)	Good CCC (n:52)	p-value
Male, n(%)	35(70)	38(73)	0.80
Age, years, mean (SD)	60.47(8.06)	59.04(8.96)	0.47
BMI, mean (SD), kg/m^2^	24.28(1.61)	24.12(1.62)	0.67
Current smoker, n (%)	15(30)	26(50)	0.11
DM, n (%)	15(30)	16(31)	0.94
Hypertension, n (%)	10(20)	13(25)	0.79
Systolic blood pressure, mean (SD), mm Hg	122.77(10.32)	125.87(11.24)	0.22
Diastolic blood pressure, mean (SD), mm Hg	74.37(8.50)	74.65(8.63)	0.88
Heart rate, mean (SD), beat/min	76.93(13.95)	76.69(13.61)	0.94
LVEF, (%), mean (SD)	55.80(8.18)	53.77(8.12)	0.28
Statins usage, n (%)	10(20)	11(21.1)	0.78
β-Blocker usage, n (%)	11(22.2)	13(25)	0.88
Nitrate usage, n (%)	3(6)	3(5.7)	0.93
Angiotensin converting enzyme inhibitor usage, n (%)	9(18)	8(15.3)	0.74
Angiotensin receptor blocker usage, n (%)	11(22.2)	7(13.5)	0.22
Calcium channel blocker usage, n (%)	10(20)	7(13.5)	0.53

*CCC: coronary collateral circulation; SD: standard deviation; BMI: body mass index; DM: diabetes mellitus; LVEF: left ventricular ejection fraction.*

The laboratory characteristics of the groups are shown in Table 2 . Mean adropine levels were found to be significantly different, in 210.83±17.76 pg/mL and 268.25±28.94 pg/mL in the poor and good CCC groups, respectively. The two groups did not differ significantly in any of the other laboratory parameters.

**Table 2:** Laboratory characteristics of the study population

	Poor CCC (n:50)	Good CCC (n:52)	p-value
Adropin level, mean (SD), pg/mL	210.83(17.76)	268.25(28.94)	<0.001
High-sensitive C-reactive protein, median (IQR), mg/L	3.55(0.93)	3.40(0.93)	0.68
Fasting glucose, mean (SD), mg/dl	117.03(33.83)	123.80(44.09)	0.48
Hemoglobin A1c, mean (SD), %	6.40(1.19)	6.39(1.02)	0.97
Total cholesterol, mean (SD), mmol/L	187.90(25.30)	194.23(28.34)	0.31
HDL, mean SD), mmol/L	43.73(5.57)	44.33(6.17)	0.67
Triglyceride, median (IQR), mmol/L	154.00(50.85)	163.50(47.20)	0.73
LDL, mean (SD), mmol/L	108.50(28.20)	110.92(29.60)	0.72
Creatinine, mean (SD), mg/dl	1.14(0.15)	1.13(0.16)	0.71
Hemoglobin, mean (SD), g/L	14.56(0.94)	14.25(1.09)	0.19
Red cell distribution width, mean (SD), %	12.79(1.01)	12.61(1.14)	0.47
White blood cell, mean (SD), x 10^9^/L	8.34(1.65)	8.36(1.44)	0.95
Neutrophil, mean (SD), x 10^9^/L	6.31(1.94)	6.21(1.44)	0.78
Lymphocyte, mean (SD), x 10^9^/L	1.75(0.44)	1.70(0.43)	0.57
NLR, mean (SD), %	3.92(1.64)	3.89(1.56)	0.76
Platelet, mean (SD), x 10^9^/L	241.90(41.35)	225.04(39.80)	0.07

*CCC: coronary collateral circulation; SD: standard deviation; NLR: neutrophil-to-lymphocyte ratios.*

Coronary angiographic characteristics of the patient groups are shown in Table 3 . No significant difference was found between the groups according to the location of the occluded coronary arteries. Mean Gensini scores of the poor and good CCC groups were found to be significantly different, in 104.3±18.9 and 95.3±14.4, respectively. There was also no difference between the groups in terms of left main coronary artery disease, multivessel disease, and bifurcation lesions.

**Table 3:** Coronary angiographic findings of the study population

	Poor CCC (n:50)	Good CCC (n:52)	p-value
LAD occlusion, n(%)	15(30)	12(23.1)	0.51
Cx occlusion, n(%)	15(30)	16(30.8)	0.93
RCA occlusion, n(%)	18(36)	24(46.2)	0.44
Gensini score, mean (SD)	104.3(18.9)	95.3(14.4)	0.007
Left main coronary artery disease, n(%)	3(6)	2(3.8)	0.08
Multivessel disease, n(%)	23(46)	21(40.4)	0.65
Bifurcation lesions, n(%)	10(20)	8(15.4)	0.14
Rentrop Score 0, n(%)	18(36)		
Rentrop Score 1, n(%)	32(64)		
Rentrop Score 2, n(%)		31(59.6)	
Rentrop Score 3, n(%)		21(40.4)	

*CCC: coronary collateral circulation; LAD: left anterior descending artery; Cx: circumflex artery; RCA: right coronary artery.*

No significant correlation was found between the adropine levels and the BMI values, heart rates, high-sensitive C-reactive protein levels, hemoglobin A1c (glycated hemoglobin) levels, smoking status, presence of DM, presence of hypertension, total cholesterol, HDL, triglyceride and LDL levels. A significant and moderately positive correlation was observed between the adropin levels and the neutrophil-to-lymphocyte ratios (NLR), whereas a significant and strongly positive correlation was observed between the adropin levels and the Rentrop scores. By contrast, a significant and moderately negative correlation was observed between the adropin levels and the age and Gensini scores ( Table 4 ) ( Figure 1 ).

**Table 4:** Correlation between adropin level and other variables of the study population

	r	p-value
Age	-0.23	0.01
BMI	-0.10	0.55
Heart rate	0.12	0.43
High-sensitive C-reactive protein	0.04	0.84
Hemoglobin A1c	0.69	0.56
Current smoker	0.33	0.16
DM	0.06	0.85
Hypertension	0.09	0.51
Total cholesterol	0.10	0.81
HDL	-0.14	0.45
Triglyceride	0.25	0.34
LDL	0.09	0.76
NLR	0.17	0.04
Gensini score	-0.19	0.02
Rentrop score	0.76	<0.001

*BMI: body mass index; DM: diabetes mellitus; NLR: neutrophil-to-lymphocyte ratios.*

**Figure 1:** Dispersion graphs showing the relationship between adropin level and a) Age (r: -0.23, p: 0.01); b) Gensini score (r: -0.19, p: 0.02); c) Rentrop score (r: 0.76, p: <0.001); d) NLR (r: 0.17, p: 0.04).

ROC curve analysis was performed to assess the role of adropin level in predicting good CCC ( Figure 2 ). The ROC analysis revealed that a cut-off value of 276.25 pg/mL in terms of adropin level predicted good CCC with 91% sensitivity and 96% specificity (ROC area=952, p<0.001).

**Figure 2:** Receiver–operating characteristic (ROC) analysis for adropin level to predict good coronary collateral circulation.

As shown in Table 2 , adropin levels were higher in the good CCC group, hence logistic analyses were performed in order to determine whether or not adropin levels can be used as an independent predictor of developing good CCC. The results of the univariate logistic regression analysis indicated that adropin levels were a strong independent predictor of developing good CCC. Gensini score, multivessel disease, LAD occlusion, and RCA occlusion were found to be independent predictors of developing good CCC as well. In addition, the results of the multivariate logistic regression analysis, which was adjusted for possible confounding factors, such as age, BMI, heart rate, total cholesterol, and low-density lipoprotein (LDL), revealed that not only the adropine level, but also the gensini score, multivessel disease, LAD occlusion, and RCA occlusion were independent predictors of developing good CCC ( Table 5 ).

**Table 5:** Univariate and multivariate logistic regression analysis showing independent predictors of good CCC

	Univariate		Multivariate*	
	p-value	OR (95% CI)	p-value	OR(95% CI)
Adropin level	<0.001	1.12(1.06-1.18)	<0.001	1.13(1.06-1.19)
Age	0.48	1.01(0.97-1.04)	0.54	1.01(0.97-1.05)
BMI	0.38	1.24(0.76-2.02)	0.45	1.23(0.74-2.01)
Heart rate	0.43	1.03(0.98-1.05)	0.51	1.04(1.01-1.07)
High-sensitive C-reactive protein	0.19	0.58(0.26-1.30)	0.41	0.61(0.30-1.42)
Hemoglobin A1c	0.96	0.98(0.51-1.90)	0.34	1.01(0.56-1.96)
Current smoker	0.12	1.86(0.89-3.89)	0.48	1.35(0.55-3.32)
DM	0.81	1.09(0.55-2.14)	0.87	1.06(0.51-2.25)
Hypertension	0.89	0.96(0.48-1.90)	0.80	0.91(0.43-1.93)
Total cholesterol	0.09	1.42(1.10-1.83)	0.10	1.44(1.09-1.90)
HDL	0.48	1.56(0.46-5.32)	0.85	1.05(0.27-4.20)
Triglyceride	0.10	1.23(0.96-1.58)	0.27	1.19(0.88-1.60)
LDL	0.77	0.99(0.97-1.02)	0.23	1.01(0.98-1.03)
NLR	0.23	0.66(0.44-1.10)	0.31	0.74(0.51-1.33)
Gensini score	<0.001	1.02(1.01-1.03)	<0.001	1.01(1.00-1.02)
Multivessel disease	<0.001	2.63(1.68-4.14)	<0.001	2.45(1.53-3.93)
LAD occlusion	<0.001	4.59(2.13-9.90)	<0.001	4.73(2.08-10.70)
Cx occlusion	0.09	2.21(1.07–4.34)	0.11	2.41(1.12-4.41)
RCA occlusion	0.01	2.31(1.17-4.53)	0.03	2.17(1.03-4.56)

*Adjusted for age, heart rate, BMI, total cholesterol and LDL. In this statistical analysis, adropin levels, as well as age, BMI, total cholesterol, HDL, triglyceride, LDL, multivessel disease, and gensini score are continuous values, others are binary variables. BMI: body mass index; DM: diabetes mellitus; NLR: neutrophil-to-lymphocyte ratios; LAD: left anterior descending artery; Cx: circumflex artery; RCA: right coronary artery.

## Dıscussıon

This is the first study in which the relationship between the adropin levels and CCC was investigated in patients diagnosed with CCS. The main finding of the study was that the adropine levels were lower in the poor CCC group than in the good CCC group. Additionally, a positive correlation was found between the adropin levels and the NLR values and Rentrop scores, whereas a negative correlation was found between the adropin levels and the age and gensini scores. Furthermore, logistic regression and ROC analyses indicated that adropin was an independent predictor of developing good CCC. Apart from the adropin level, other factors such as Gensini score, presence of multivessel disease, LAD occlusion, and RCA occlusion have been shown to be predictive of developing good CCC as well.

CCC occurs when the coronary vessels narrow down for 70% or more.^14^ The resultant collateral vessels are between 20-200 µm in size and are thin-walled. The denseness of the collateral vessels formed varies from species to species, and it is moderate in humans.^15^ These vessels are the alternative blood supply pathway of the ischemic myocardium. CCC vessels are normally closed and non-functional. However, when the pressure difference occurs as a result of coronary stenosis, the rudimentary vessels rapidly open up.^14^

Coronary collateral arteries help maintain myocardial functions by providing an alternative blood flow to myocardial tissue left ischemic by occlusive CAD. It is usually ischemia that gives rise to an excess of collateral arteries, yet even those without CAD have an excess of collateral arteries, as the existing CCC may render insufficient during exercise even though it provides the blood needed by the myocardium while at rest. Several independent clinical and angiographic variables have been associated with the CCC grade in the literature. In patients with CAD, the time of occlusion,^16^ the location of the lesion, the severity of coronary stenosis, and the duration of angina^17^ affect the degree of CCC; while in healthy individuals, hypertension and resting heart rate^18^ affect the degree of CCC.

The clinical importance of CCCs is that they protect the myocardial functions,^19^ limit the infarct size^20^ and positively affect ventricular remodeling,^21^ particularly during the acute myocardial infarction. Additionally, it has been also reported in the literature that CCCs partially reduced the incidence of concomitant cardiogenic shock.^22^

Recently, it has been suggested that the most important factors in the development of CCC are the production of endothelium-derived NO and VEGF. It is known that NO and VEGF increase angiogenesis, especially in coronary collateral vessels, and contribute to the maturation of collateral arteries.^23^ Adropin has been shown to increase VEGFR-2 in endothelial cells, and as a result, it has also been shown to increase the expression of eNOS mRNA and eNOS protein, via Akt (Ak strain transforming), that is protein kinase B, and ERK½ (extracellular signal-regulated protein kinase ½) as well.^8^ Thus, it is obvious that coronary collaterals will mature further through VEGFR-2. As a matter of fact, in this clinical study, a positive and significant correlation was observed between the Rentrop scores, which indicate the coronary collaterals, and the adropin levels, substantiating the findings of the above-mentioned cellular study.

A relationship has been shown between CAD and low adropin levels; and SYNTAX (SYNergy between percutaneous coronary intervention with TAXus and cardiac surgery), Gensini and Friesinger scores and serum adropin levels were shown to be negatively correlated in the patient group with type 2 DM.^24^ It has been suggested that low serum adropin levels are an independent predictor of coronary atherosclerosis^24^ and the patency of saphenous vein grafts after CABG operation.^25^ In comparison, in this study, similar to the findings of other studies mentioned above, a moderately negative correlation was found between the Gensini scores and the adropin levels. Nevertheless, in this study, patients with type 2 DM comprised 30.5% (30% in the poor CCC group, 31% in the good CCC group) of the study group. In addition, the patients, who underwent CABG, were not included in this study.

Several studies have suggested that there is an inverse relationship between aging and adropin levels, and that this decrease in adropin levels may be one of the minor factors that trigger CAD, which is known to increase with age.^9 , 24^ It was also shown in another study that the effect of adropin-induced eNOS-mediated vasodilation decreases with age.^26^ In comparison, in this study, similar to the findings of other studies mentioned above, a significant moderate correlation was observed between adropin levels and age in the negative direction.

There is no doubt that the NLR is associated with inflammation and that the inflammation plays a role in CAD. To give an example, in a study conducted with chronic CCS patients, mean NLR was found as 5.0±5.1 in the group with atherosclerosis progression, and as 3.2±3.0 in the group without progression, and this finding was attributed to the correlation between the atherosclerosis progression and increased NLR.^27^ In addition to the associated classical risk factors, NLR has been shown to be associated with the prevalence of CAD and the complexity of the lesions as well.^28^ In another controlled study, high NLR values proved to be a good predictor of Gensini scores in the group of patients with CCS. NLR values above 2.04 were found to have effectively predicted the presence of CAD.^29^ In fact, it has been shown in another study that NLR values even predicted the chronic total occlusions of patients.^30^ A correlation was reported between good CCC development and NLR in the group of patients with concomitant chronic total occlusion.^31^ In comparison, in this study, contrary to the respective findings reported in the literature, NLR was not found to have differed between the poor and good CCC groups, yet it was found to have correlated with adropin levels. It is thought that this discrepancy between the said result of this study and the respective results reported in the literature might be due to the low number of patients included in this study.

### Limitations to the study

There were several limitations to this study. First, the number of patients included in this study was limited, and secondly, there was no control group comprising individuals with normal coronary arteries. Hence, it would be beneficial to replicate the study with a larger study group and with the addition of a control group. Additionally, the development of CCC is a long process, thus a single measurement of adropin levels may not give a clear idea about lifelong development of CCC. Another limitation was that Rentrop classification, a visual method used in the evaluation of CCC, was used, and intravascular ultrasonography was not used. CCC examined in the Rentrop classification are affected by the patient’s blood pressure, the contrast injection strength of the operator, and the filming time. Lastly, despite the fact that a correlation was found between the adropin levels and the CCC, the underlying mechanisms are not clear, thus large-scale studies are needed to verify the effect of adropin on the development of CCC.

## Conclusion

In conclusion, the findings of this study suggest that adropin levels correlate with the presence and amount of CCC in CCS patients.
